# Neat plasma proteomics: getting the best out of the worst

**DOI:** 10.1186/s12014-024-09477-6

**Published:** 2024-03-12

**Authors:** Ines Metatla, Kevin Roger, Cerina Chhuon, Sara Ceccacci, Manuel Chapelle, Pierre-Olivier Schmit, Vadim Demichev, Ida Chiara Guerrera

**Affiliations:** 1 Proteomics Platform Necker, Université Paris Cité-Structure Fédérative de Recherche Necker, INSERM US24/CNRS UAR3633, 75015 Paris, France; 2 Bruker Daltonique SA, 34 Rue de l’Industrie, 67166 Wissembourg Cedex, France; 3 Department of Biochemistry, Charité-Universitätsmedizin Berlin, 10117 Berlin, Germany

**Keywords:** Plasma proteomics, diaPASEF, Neat plasma, Extracellular vesicles (EVs), DIA-NN

## Abstract

Plasma proteomics holds immense potential for clinical research and biomarker discovery, serving as a non-invasive "liquid biopsy" for tissue sampling. Mass spectrometry (MS)-based proteomics, thanks to improvement in speed and robustness, emerges as an ideal technology for exploring the plasma proteome for its unbiased and highly specific protein identification and quantification. Despite its potential, plasma proteomics is still a challenge due to the vast dynamic range of protein abundance, hindering the detection of less abundant proteins. Different approaches can help overcome this challenge. Conventional depletion methods face limitations in cost, throughput, accuracy, and off-target depletion. Nanoparticle-based enrichment shows promise in compressing dynamic range, but cost remains a constraint. Enrichment strategies for extracellular vesicles (EVs) can enhance plasma proteome coverage dramatically, but current methods are still too laborious for large series. Neat plasma remains popular for its cost-effectiveness, time efficiency, and low volume requirement. We used a test set of 33 plasma samples for all evaluations. Samples were digested using S-Trap and analyzed on Evosep One and nanoElute coupled to a timsTOF Pro using different elution gradients and ion mobility ranges. Data were mainly analyzed using library-free searches using DIA-NN. This study explores ways to improve proteome coverage in neat plasma both in MS data acquisition and MS data analysis. We demonstrate the value of sampling smaller hydrophilic peptides, increasing chromatographic separation, and using library-free searches. Additionally, we introduce the EV boost approach, that leverages on the extracellular vesicle fraction to enhance protein identification in neat plasma samples. Globally, our optimized analysis workflow allows the quantification of over 1000 proteins in neat plasma with a 24SPD throughput. We believe that these considerations can be of help independently of the LC–MS platform used.

**Supplementary Information:**

The online version contains supplementary material available at 10.1186/s12014-024-09477-6.

## Background

Plasma proteome holds extraordinary promises for advancing clinical research and biomarker discovery, representing an easily accessible and minimally invasive “liquid biopsy” potentially enabling sampling of tissues [1, 2]. Mass spectrometry (MS)-based proteomics, due to its unbiased nature as well as its high specificity in identifying and quantifying proteins and proteoforms, is the ideal technology for investigating the plasma proteome [3]. The remarkable potential of plasma is counterbalanced by its formidable complexity, making it the most challenging biofluid for proteomics analysis [4].

The main analytical challenge is the large dynamic range of plasma protein abundance, spanning over 12 orders of magnitude [5]. This limits the identification of protein biomarkers: indeed, the 22 most abundant proteins, collectively constituting 99% of plasma protein mass, with albumin accounting for 50%, prevent the detection of less abundant proteins. Depletion of the most abundant plasma proteins can be performed to partially decrease the dynamic range [6]. Nevertheless, conventional depletion techniques like immunoaffinity subtraction chromatography and extensive fractionation have inherent limitations including increased costs, reduced throughput, compromised accuracy in protein quantification, and off-target depletion due to carrier functions of many abundant proteins [1, 7]. Furthermore, even after immunodepletion, the most abundant plasma proteins can still make up 90% of the total signal in a shotgun proteomics analysis [8]. Nanoparticle based enrichment holds great promise in dynamic range compression leading to a five to ten increase in the coverage of plasma soluble proteome [9–11]. The high cost of these recent technologies is still a limit for a more widespread implementation.

Enrichment strategies for extracellular vesicles (EVs) from plasma can significantly increase the number of features identified in the plasma, up to 4000 proteins [12]. However, established EVs enrichment for proteomics analysis although cost-effective are laborious and time-consuming, hence not applicable to cohort studies. New promising technology based on magnetic beads are emerging, but they still have to be proven robust [13]. Furthermore, EVs plasma proteomics leads to enhanced identification of intracellular rather than soluble proteins, which are most commonly used as biomarkers.

As a result, neat or non-depleted plasma has retained popularity in clinical analysis primarily due to its cost-effectiveness, time efficiency and the low initial volume requirement [14].

Additional challenges related to high variability arise when conducting any plasma proteomics study. Firstly, the preanalytical variability linked to plasma sample collection methods and processing techniques strongly influences the quality and consistency of plasma proteomics data [1, 4, 15]. Secondly, despite all efforts to minimize preanalytical variables, systematic bias is inherent when dealing with human samples and multi-centric cohort analysis. Separating disease-related variation from interpersonal variability, due to biological and environmental factors, is particularly challenging in plasma and requires increasing sample size and enhancing the depth of analysis [1].

Data Independent Acquisition (DIA) has significantly increased plasma proteome coverage, limiting inherent issues related to missing values and limitations in quantification accuracy associated with Data Dependent Analysis (DDA). In fact, as reported in recent studies, DIA outperforms DDA by a factor of two, allowing the identification of up to 600 proteins with LC gradients of 60–90 min and around 300–400 proteins in high-throughput analysis employing shorter separation times of under 20 min [14]. diaPASEF, which combines DIA with ion mobility separation [16, 17], has contributed to enhance robustness, throughput and depth in plasma proteomics analysis [18]. Since the peculiar nature of plasma proteome [19], optimizations of LC–MS methods can potentially increase plasma proteome coverage.

In this study, we share some observations and tips to improve the detection of proteins in neat plasma. We assessed the value of sampling at lower IM and m/z ranges using diaPASEF-MS, the importance of higher chromatographic separation, and the impact of running library-free searches over a large number of samples. Furthermore, we provide evidence of the advantage of using the extracellular vesicle fraction as a repertoire to boost the identification of proteins in neat plasma samples.

## Methods

### Plasma collection and preparation

Optimization tests have been performed using healthy volunteers’ plasma. For the small-sized clinical study we used a plasma sample cohort consisting of samples from 15 patients affected by a rare dermatological genetic disease (RDGD) and 18 age-matched controls patients (CP). Healthy volunteers and RDGD patients provided informed consent. Peripheral blood was collected in EDTA tubes. Plasma (2 mL) from healthy volunteers was obtained from peripheral blood by centrifugation twice at 1500 g for 10 min. Plasma (2 mL) from RDGD and control patients was obtained from peripheral blood by centrifugation at 2000 g for 5 min. All samples were immediately stored at –80 °C.

### EV preparation

Extracellular vesicles were obtained by a centrifugation method based on the protocol published by Geiger’s laboratory [20, 21]. All the steps were carried out at 4◦C. Briefly, plasma was centrifuged at 3300 × g for 20 min to remove platelets. Supernatants were collected in clean tubes and diluted twofold with ice-cold PBS and centrifuged at 20,000 × g for 1 h. Pelleted extracellular vesicles were then washed twice with the same volume of ice-cold PBS and suspended in 5% SDS, 50 mM triethylammonium bicarbonate (TEAB).

### Protein digestion

S-Trap™ micro spin column (Protifi, Hutington, USA) digestion was performed on 1µL of plasma according to manufacturer’s instructions. Briefly, samples were supplemented with 20% SDS to a final concentration of 5%, reduced with 20 mM TCEP (Tris(2-carboxyethyl) phosphine hydrochloride) and alkylated with 50 mM CAA (chloracetamide) for 5 min at 95 °C. Aqueous phosphoric acid was then added to a final concentration of 2.5% followed by the addition of S-Trap binding buffer (90% aqueous methanol, 100 mM TEAB, pH7.1). Mixtures were then loaded on S-Trap columns. Four washes were performed for thorough SDS elimination. Samples were digested with 2.5 µg of trypsin (Promega) at 47 °C for 1 h. EVs pellets were resuspended in 25µL of SDS 5% in 50 mM TEAB with reducing and alkylating reagents for a final concentration of 20 mM TCEP and 50 mM CAA. Then, EVs were heated at 95 °C for 5 min. S-Trap™ micro spin column was performed on the 25µL according to manufacturer’s instructions. An amount of 2.5 µg of trypsin at 47 °C for 2 h was used to digest the proteins. After elution, peptides were vacuum dried.

### Nano-LC–MS/MS protein identification and quantification

Peptides were resuspended in 2% ACN, 0.1% formic acid in HPLC-grade water and 200 ng were injected on a nanoelute (Bruker Daltonics, Germany) HPLC (high-performance liquid chromatography) or an Evosep One system coupled to a timsTOF Pro 2 (Bruker Daltonics, Germany) mass spectrometer. HPLC separation (Solvent A: 0.1% formic acid in water; Solvent B: 0.1% formic acid in acetonitrile) was carried out on the nanoElute using a packed emitter column (C18, 25 cm × 75 μm 1.6 μm) (Ion Optics, Australia) with a 12 min active gradient elution (2 to 17% solvent B during 8 min; 17 to 25% during 2 min; 25% to 37% during 2 min; 37% to 95% for 1 min and finally 95% for 2 min to wash the column, flowrate 400nL/min) or 30 min active gradient elution (2 to 11% solvent B during 19 min; 11 to 16% during 7 min; 16% to 25% during 4 min; 25% to 80% for 3 min and finally 80% for 7 min to wash the column, flowrate 250nL/min). All the gradients referred hereafter are the active gradients unless stated otherwise. The Evosep One system operated with the 100 or 60 Samples Per Day (100 SPD or 60 SPD) method using an 8 cm C18 Performance column and on a 40SPD whisper method using a packed emitter column (C18, 15 cm × 75 μm 1.7 μm) (Ion Optics, Australia). All the details of the chromatographic settings for all method used are reported in Additional file 1: Table S1.

Mass-spectrometric data were acquired using the parallel accumulation serial fragmentation (PASEF) acquisition method in DIA mode with a 19-windows method using 33 Da windows covering the mobility ranges over a 400–1050 m/z range.

For the Evotip offline experiment, Evotips Pure (n = 3) were rinsed with 20 µl Solvent B (0.1% formic acid in acetonitrile), conditioned with propanol and equilibrated with 20 µl Solvent A (0.1% formic acid in water). 200 ng of plasma sample were loaded onto each Evotip that was subsequently centrifuged at 800 g for 60 s. The resulting flow-throughs (FT) were collected and the Evotips washed with 20 µl Solvent A. After centrifugation, the wash (W) was recovered and pooled with the previous F1. Peptides were then eluted from each Evotip with 100 µl of 95% acetonitrile containing 0.1% formic acid. Finally, flow-throughs (FT + W) and eluates were dried under vacuum and resuspended in 2% acetonitrile 0.1% formic acid. 200 ng of total plasma (Input), eluates and flow-throughs (F1 + W) were analysed on nanoElute coupled to timsTOF Pro using a packed emitter column (C18, 25 cm × 75 μm 1.6 μm) (Ion Opticks, Australia) with the 24 SPD method and the Broad mass method.

### Bioinformatics data analysis

Data analysis of all DIA files was performed using DIA-NN software (version 1.8.1 and version 1.8.2 beta27 for the EV boost search) [22]. A search against the human UniProtKB/Swiss-Prot *Homo sapiens* database (downloaded in February, 2021, 20,396 entries) was performed using library free workflow. For this purpose, “FASTA digest for library free search/library generation” and “Deep learning spectra, RTs and IMs prediction” options were checked for precursor ion generation. A maximum of 1 trypsin missed cleavages was allowed and the maximum variable modification was set to 2. Carbamidomethylation (Cys) was set as the fixed modification, whereas protein N-terminal methionine excision, methionine oxidation and N-terminal acetylation were set as variable modifications. The peptide length range was set to 7–30 amino acids, precursor charge range 2–4, precursor m/z range 300–1300, and fragment ion m/z range 300–1300. To search the parent mass and fragment ions, accuracy was set to 10 ppm manually. The false discovery rates (FDRs) at the protein and peptide level were set to 1%. Match between runs was allowed.

The classical pg.matrix is a standard output file from DIA-NN and it corresponds to the main report file filtered for Q.Value, Lib.Q.Value and Lib.PG.Q.Value all set below 1% (Additional file 2: Table S2). This refers to the MBR search.

Regarding the EV-boost searches, 3 different statistical metrics present in the report file were set to a value below 1% (Q.Value, Lib.Q.Value and Lib.PG.Q.Value) in order to have the exact same filters applied to generate the pg.matrix output file automatically produced at the end of a DIA-NN run. We also used additional run-specific q-value filter for the protein group (PG.Q.Value) at 5%, 3% or 1%.

The DDA MS files were processed with the MaxQuant software version 2.3.0.0 and searched with Andromeda search engine against the UniProtKB/Swiss-Prot *Homo sapiens* database (released in February 2021, 20,396 entries). To search parent mass and fragment ions, we set a mass deviation of 10 ppm for the main search and 40 ppm respectively. The minimum peptide length was set to 7 amino acids and strict specificity for trypsin cleavage was required, allowing up to 2 missed cleavage sites. Carbamidomethylation (Cys) was set as fixed modification, whereas oxidation (Met) and N-term acetylation (Prot N-term) were set as variable modifications. The false discovery rates (FDRs) at the peptide and protein level were set to 1%. Scores were calculated in MaxQuant as described previously. The reverse and common contaminants hits were removed from MaxQuant output as well as the protein only identified by site. Proteins were quantified according to the MaxQuant label-free algorithm using LFQ intensities and protein quantification was obtained using at least 1 peptide per protein. Finally, a match between runs was allowed during the analysis.

All R figures was created using R (version 4.2.2) and RStudio (version 2022.07.2). The majority of the figures (barplot, density plot, violin-box plot, scatterplot, lineplot) were created using ggplot2 (v3.4.4) embedded inside the tidyverse (v2.0.0). For specific purposes, ggpubr (v0.6) R package was used and Venn diagram was created the ggvenn (v0.1.10). The investigation about physico-chemical properties of peptides was performed with Peptides (v2.4.5) R package.

The mass spectrometry proteomics data have been deposited to the ProteomeXchange Consortium via the PRIDE [23] partner repository with the dataset identifier PXD047857. All the protein and peptides tables, all R scripts, and plots generated to compose the figures are reported on Additional files by figure number.

## Results

### Impact of dia-PASEF acquisition method optimization

**Fig. 1:**
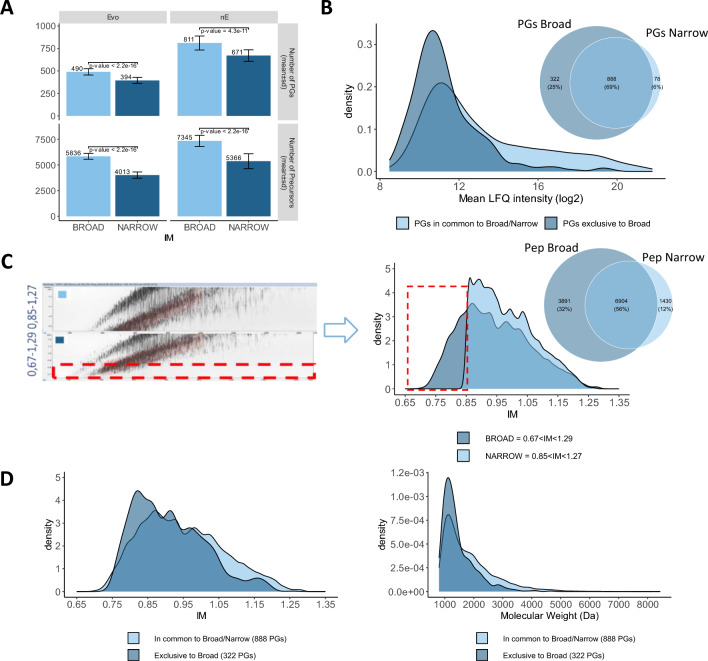
Impact of dia-PASEF acquisition method optimization. **A** Number of identified protein groups and precursors across 33 neat plasma samples (mean ± standard deviation) using “Narrow” and “Broad” methods for nE and Evosep liquid chromatography systems. Welch Two Sample t-test are included for each comparison with respective p-value. **B** Venn diagram and mean LFQ intensity distribution of the protein groups relationships between “Narrow” and “Broad” methods. **C** Global ion map (left panel), ion mobility distribution of precursors from “Narrow” and “Broad” method and corresponding Venn Diagram of their respective precursors (right panel). **D** Ion mobility (left panel) and molecular weight (right panel) distribution of the peptides exclusively found in the “Broad” method and in common between “Broad” and “Narrow” methods

We tested two different dia-PASEF acquisition methods: one method with narrower ion mobility (IM) (1/K0 = 1.27 to 0.85 Vs cm-2) and one with broader ion mobility (1/K0 = 1.29 to 0.67 Vs cm-2). Mass range was also broadened from 475–1000 Da to 400–1027 Da. The “Broad” method allows to significantly identify 21% to 24% more proteins and 37% to 45% peptides on average (Fig. 1A), suggesting that the ions with low ion mobility, between 0.67 and 0.85 Vs cm-2, contain additional peptides valuable for lower abundance protein identification (Fig. 1B, 1C).

To corroborate this observation, we analyzed the distribution of ion mobility for the peptides attributed to the proteins identified with the “Broad” method (1,210 protein groups, 10,795 peptides in total respective matrices), with the “Narrow” method (966 protein groups, 8,334 modified sequences in total respective matrices) (Fig. 1C). We also compared the IM and MW distribution of the peptides identified in the “Broad” (3,891) to the distribution of the peptides found in common between the two methods (6,904) (Fig. 1D, Additional file for Fig. 1). We observe that peptides derived from the proteins (322) identified exclusively with the “Broad” method have a distribution of ion mobility and molecular weight shifted towards lower values. This proves that broadening of the ion mobility and mass range effectively contributes to increasing the number of PG IDs in neat plasma mixture by sampling additional peptides in the low mass range.

Based on these results, we decided to use the “Broad” acquisition method to continue the evaluations.

### Impact of peptide RPLC separation

With the aim to reduce the overall run time, we compared different gradient lengths on the Evosep One 100 SPD (11 min gradient) and 60 SPD (21 min gradient) using an 8 cm Evosep column as well as on the nanoElute (12 and 30 min gradient) using a 25 cm emitter embedded IonOpticks column. Although a 25 cm column is not optimal for the 12 min gradient, we kept the same column for both methods, as it allowed us to evaluate the influence of the effective gradient length for plasma samples analysis on the nanoElute, independently of the analytical column. Given the overhead time due washing and equilibration, the effective throughput for these methods was 48 SPD and 24 SPD, respectively.

**Fig. 2:**
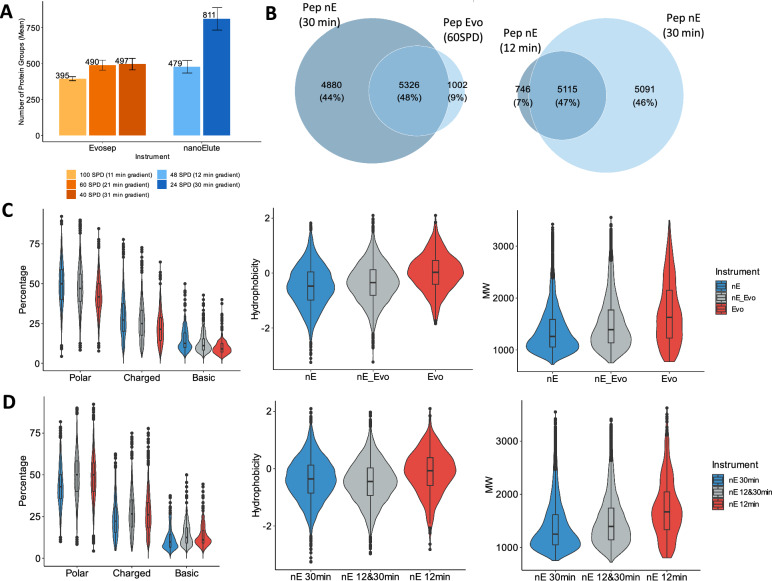
Impact of peptide separation: column length and gradient influence. **A** Number of identified protein groups across 33 neat plasma samples (mean ± standard deviation) using “Broad” methods with different liquid chromatography setups (instruments, columns, gradients). **B** Venn diagram of the peptides relationship between: left panel: nE 24SPD (30 min gradient) and Evo 60 SPD (21 min gradient); right panel: nE 48SPD (12 min gradient) and nE 24SPD (30 min gradient). **C** Physico-chemical properties of peptides from the 3 Venn Diagram groups of B (left panel).** D** Physico-chemical properties of peptides from the 3 Venn Diagram groups of B (right panel)

As shown in Fig. 2A, using the shorter gradients significantly reduces the number of proteins identified. The number of PG IDs using the 12 min gradient with the 25 cm column (average of 479 ± 44 PG IDs, 48 SPD) is comparable to the number of PG IDs obtained with the 60 SPD method (490 ± 35 PG IDs, 60 SPD) allowing a better throughput. Reducing the gradient even further to 11 min (100 SPD) led to 20% less in PG IDs for a 66% increase of throughput compared to 60 SPD. The set-up leading to a significant increase in PG IDs compared to all other settings tested, is the separation over 30 min gradient with the 25 cm column, allowing the identification of an average of 811 ± 78 PG IDs in 1 h global run time (24 SPD).

In order to understand the nature of the additional peptides identified using a longer gradient on the nanoElute, we analyzed the physicochemical properties of the peptides exclusively identified by nanoElute 30 min gradient (10,206 peptides in total, of which 4,880 exclusive) compared to analysis of the same samples using shorter gradients 60 SPD (Evosep One, 6,328 peptides, 1,002 exclusive peptides) and 48 SPD (nanoElute, 5,861 peptides, of which 746 exclusive).

As reference, we used peptides identified in common for each comparison (5,326 and 5,115 peptides respectively for 60 SPD Evosep One and 48 SPD nanoElute) (Fig. 2B and C), (Additional file 3: Fig. S1 for all properties).

We observe that peptides exclusively identified using longer gradient, with a mild initial gradient, were higher in average number of polar and charged amino acids while 60 SPD and 48 SPD exclusive peptides have lower average number of basic AA (K, R, H), suggesting that this analysis favors the detection of shorter peptides. To corroborate this observation, we compared the average MW and hydrophobicity of the peptides and we found they were both higher in 60 SPD and 48 SPD specific peptides compared to peptides in common and even more compared to 24 SPD peptides (Fig. 2C and D, Additional file 4: for Fig.S2).

To understand if the loss of peptides is linked to the use of the precolumn on the Evosep, we performed off-line elution on the Evotip. Our data provides evidence that the use of Evotips exerts negligible impact on the number of peptides and proteins identified and is not the main source of the loss of hydrophilic peptides in the analysis (Additional file 3: Fig. S1).

These data suggest that both the standard Evosep One 60 SPD and the default 48 SPD analysis using 25 cm analytical columns do not favor the detection of hydrophilic peptides which are crucial to additional protein identifications. Separating the peptides on Evosep One using a 15 cm columns and a longer active gradient of 31 min still did not improve the results (standard Evosep 40 SPD whisper method), (Fig. 2A).

### Impact of the data search strategy

**Fig. 3:**
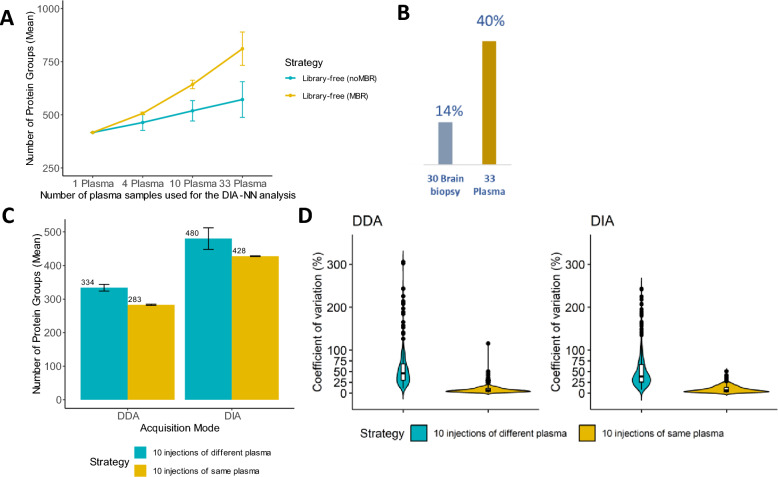
Impact of data search strategy. **A** Number of identified protein groups across 1, 4, 10 and 33 neat plasma samples (mean ± standard deviation) analyzed with DIA-NN v1.8.1 library-free search strategy with and without Match Between Runs (MBR). **B** Number of identified protein groups across 30 brain biopsy compared with 33 neat plasma (DIA-NN v1.8.1 with MBR). **C** Number of identified protein groups across 10 injections of different neat plasma (biological variability) and 10 injections of same plasma (technical variability) (mean ± standard deviation) in DDA and DIA acquisition modes (Evo-TTP) and analyzed with DIA-NN v1.8.1 library-free search strategy (with MBR) and Maxquant v2.3.0.0 (with MBR)**. D** Coefficient of variation distribution calculated from LFQ raw intensities (non log transformed) across 10 injections of different neat plasma (biological variability) and 10 injections of same plasma (technical variability) in DDA and DIA acquisition modes and analyzed with DIA-NN v1.8.1 library-free search strategy (with MBR) and Maxquant Maxquant v2.3.0.0 (with MBR)

We further evaluated the impact of Match Between Run (MBR) on the depth of the analysis. For this, we employed DIA-NN in library free mode, with or without Match Between Run (MBR). We observed an increase of PG IDs as more sample files are searched at the same time, in particular when MBR was allowed (Fig. 3A). This phenomenon is expected, but it is significantly accentuated in plasma samples compared to patients’ tissue samples: searching 33 plasma sample files together allowing MBR, led to an increase of PG IDs by 40%, whereas using 30 brain lysate sample files, lead to an increment of only 14% (Fig. 3B).

The MBR concept is based on on-the-fly creation of a spectral library from the DIA experiment, with subsequent highly sensitive re-search of all the samples using this library. The main benefits of MBR manifest in experiments with samples heterogeneous in sample loading, with information obtained from acquisitions where a peptide is confidently identified being used to boost its detection in other acquisitions, improving proteomic depth and data completeness [22, 24, 25]. In order to quantify the interpersonal proteome variability in blood plasma, we measured 10 plasma samples from 10 individuals both in DIA and DDA mode, and compared it to 10 runs of the same plasma (technical replicates). In DIA mode, we observe a median CV of 37% across all proteins in biological replicates as opposed to 6% in technical replicates (10 runs of the same plasma). Results obtained using DDA acquisition showed very similar results with CVs of 46% and 6% respectively (Fig. 3C and D, Additional file 5: Fig. S3 for Fig. 3). These results are in line with observations previously made on different LC–MS systems as well [26]. This confirms that the interpersonal variability in plasma protein abundance is very high across different individuals and we suggest that it may be the main contributing factor to the positive effect of MBR using a library-free approach.

Plasma sample contamination from blood cells can lead to an increased number of proteins ID because of the identification of additional proteins derived from platelets. This will depend on the protocol of collection, and it could typically happen when platelet rich plasma (PRP) is used. We verified that the average number of proteins was the same whether we used PRP samples or platelet poor plasma (PPP), and that the level of known contaminants was similar. This contamination was measured according to http://plasmaproteomeprofiling.com/, which provides a ratio between typical contaminants and plasma proteins [15]. Only three samples contained a high contamination ratio (sample N3, N4, N9) and uniquely for sample N3 the contamination with red blood cells (RBC) correlated with a higher number of protein IDs (Additional file 4: Figure S2).

### Plasmatic extracellular vesicles as a repertoire of plasma proteins

Extracellular vesicles (EV) are secreted by all cell types and are found in biofluids. In plasma, they are mainly derived from plasma cells. EV contain biological material from the generating cells, including intracellular proteins and plasma proteins on their surface [12, 27–29]. EVs are therefore a biological fraction of the neat plasma rich in information.

**Fig. 4:**
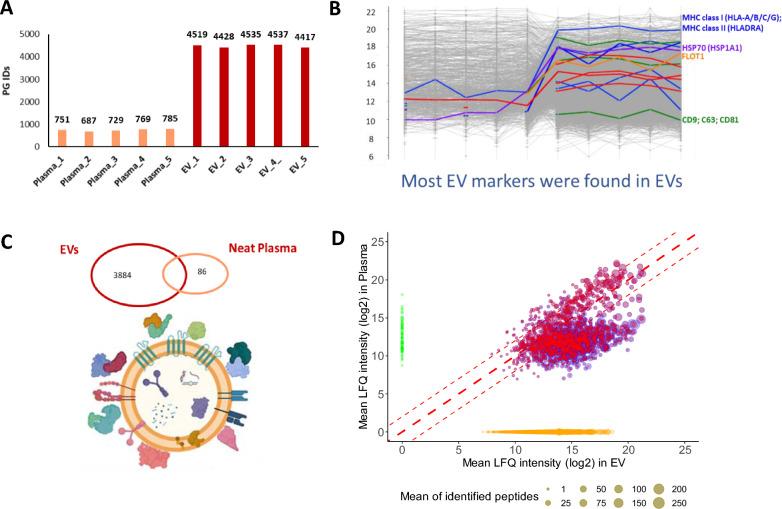
Plasmatic extracellular vesicles as a repertoire of plasma proteins; **A** Number of identified protein groups in 5 neat plasma samples and in 5 EVs purified from the same plasma samples. **B** Known EV markers uniquely identified in EV fractions or enriched in them. **C** Venn diagram of the protein groups relationship between protein groups identified in neat plasma and EV fractions. **D** Correlation plot between mean LFQ intensity of proteins exclusively found in plasma (light green) or in EV fraction (orange) or in common in both (gradient color from blue to red according to number of identified peptides)

To compare the proteome of the EV fraction with the total plasma proteome, we prepared the EV fraction through multiple centrifugation steps, as previously described [20, 21]. We also processed neat plasma from the same samples. An average of 4,487 proteins could be identified in the EV fractions from plasma obtained from five different individuals, compared to an average of 744 proteins when the same samples were analyzed as neat (Fig. 4A, Additional file for Fig. 4).

Most of the known EV markers could be identified uniquely or were strongly enriched in the EV fractions (Fig. 4B). Interestingly, 90% of the proteins identified in the neat plasma analysis were also identified in the EV fractions (Fig. 4C). A correlation plot of the intensity of the proteins found in both fractions shows a good correlation of their abundance. In particular, there is a subset that is highly correlated and a subset of proteins that are enriched in the EV fraction (Fig. 4D).

These data imply that proteins identified in the plasmatic EV fraction include core EV proteins and soluble proteins associated with the EV, and that they may constitute a rich repertoire of plasmatic proteins.

### Plasmatic extracellular vesicles as a reference in the neat plasma search

Given the positive impact of Match Between Runs (MBR) in the direct neat plasma searches, we explored the feasibility of extending the MBR to EV files using DIA-NN. Instead of building a peptide library, we integrated raw files from EV samples alongside the files from neat plasma samples. We enabled MBR and conducted a library-free search. In this manner, MBR was allowed within the neat plasma samples, as shown above (refer to Fig. 3), and also between neat plasma files and EV files. We refer to this search approach as the ‘EV-boost search’.

Using MBR amongst sample of different nature, such as neat plasma and plasma EVs raises reasonable questions on the false discovery rate control. We assessed the EV-boost search using DIA-NN. We evaluated the number of identified proteins reported in the protein group output table and the number of proteins obtained applying various filters of global and run-specific precursor/protein q-values to the main peptide table (see Materials and method section).

**Fig. 5:**
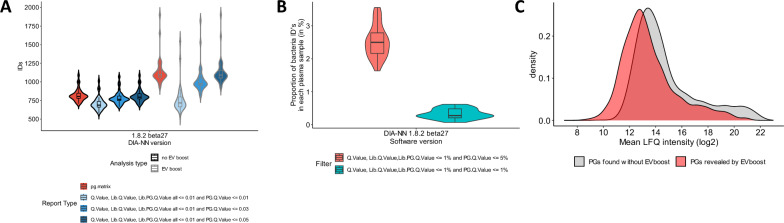
Plasmatic extracellular vesicles as a reference in the neat plasma search. **A** Distribution of identified protein groups across 33 neat plasma with and without EV boost search. For each analysis, different filters were applied in DIA-NN 1.8.2 beta27, particularly when EV boost search was applied in order to assess confidence in identification. **B** Density plots of plasma proteins identified in at least 1 neat plasma sample (3035 over 5030 PGs in total matrix) when EV boost search is applied and analyzed with DIA-NN 1.8.2 beta27. **C** In grey are represented the distribution of the plasma proteins found without EV boost and in red are represented the distribution of the newly identified proteins (1841) thanks to EV boost

Without EV-boost search, we observed a median PG IDs of 804 across all 33 neat plasma files using the protein group table (Fig. 5A, black edge, red background), very similarly to report matrix with an additional filter set at 5% on the run-specific q-value for the protein group (median PG IDs at 794) (Fig. 5A, black edge, dark blue background). We applied the same stringencies to EV-boost search in order to have confident IDs using MBR with very different sample types (plasma and EVs). With EV-boost search, we observe a median PG IDs of 1,095 across all 33 neat plasma files using the protein groups table(Fig. 5A, gray edge, red background) and a median PG IDs at 1,092 using the report matrix with run-specific q-value for the protein group set at 5%. These data indicate that the EV boost search allows an increase of 38% in terms of median PG IDs (794 to 1,092) (with run-specific protein q-values at FDR 5%).

We investigated the distribution of the proteins identified in the neat plasma files and highlighted the additional proteins ID distribution obtained using the EV-boost in red (Fig. 5C). Proteins identified uniquely by EV-Boost show a global distribution shifted toward lower abundances.

## Discussion

Plasma is probably the most challenging biological sample to analyze using proteomics. Yet, because of its specificity and unbiased search for proteoforms, plasma proteomics is one of the most powerful analytical approaches for clinical measurements and biomarker research. Unprocessed or neat plasma, despite its complexity, holds practical advantages: it’s cost-effective, scalable, automatable, and demands only a minimal one-microliter plasma sample.

Based on our results, we share three observations that can help improve neat plasma proteome analysis.

First, we show that sampling peptides which are smaller in size and more hydrophilic leads to an increase of protein groups identified in plasma. Plasma proteome and cellular proteome are bound to differ in terms of the physicochemical properties of the peptides generated by trypsin digestion. For example, the plasma proteome is enriched in soluble proteins, which tend to be more hydrophilic than cellular proteins involved in cell structure or membranes. As a result, peptides generated from plasma proteins by trypsin digestion are likely to be more hydrophilic as well. The difference in physicochemical properties between peptides from plasma and cellular proteins can impact their chromatographic behavior as hydrophilic peptides are more likely to elute early from a reversed-phase chromatography column, while hydrophobic peptides are more likely to elute late. Our work shows that the optimization of RPLC conditions and MS parameters to sample hydrophilic peptides can improve the plasma proteome coverage.

We used two liquid chromatography (LC) systems: EvosepOne and nanoElute. Evosep is a highly standardized LC system, with unmodifiable methods, that ensures remarkable robustness and true high-throughput. For these reasons, it is the usual choice for the analysis of large cohorts of samples. NanoElute LC is a classic high-performance liquid chromatography (HPLC) system, with greater flexibility in the choice of chromatographic columns and gradients. Our data show that a longer gradient on a nanoElute system with a slow initial gradient, coupled with high-resolution columns, delivers significantly better results than shorter standard analysis. We show that the increased number of proteins identified is directly associated with a higher number of hydrophilic peptides detected using the longer gradient. We observed that shorter gradients lead to a loss of these peptides, independently of the LC system used. We show that the detection of hydrophilic peptides is more associated with the LC resolution and separation time than the small loss of these peptide through the Evotip.

Following the same principle, and in line with these observations, setting the MS method to sample ions at lower m/z and lower ion mobility allows recovery of additional peptides which are informative for protein identification. We therefore suggest adjusting MS parameters to include these smaller ions and optimizing the LC system to detect low m/z ions will improve the results of neat plasma analysis on any LC–MS platform.

Second, we show that data analysis can leverage intrinsic interpersonal variability. Match-between-runs (MBR) is a powerful mode in DIA-NN that enables the software to match peptides across multiple DIA runs. This can be useful for improving the depth of coverage and accuracy of protein identifications, especially for samples with low protein concentrations or for experiments with multiple replicates. MBR works by first creating a spectral library from one of the DIA runs. This library is then used to search the other DIA runs. When a peptide is identified in a DIA run, DIA-NN will check to see if it is also present in the spectral library. If it is, DIA-NN will use the spectral library to improve the accuracy of the peptide identification. We clearly show that by analyzing up to 33 plasma files simultaneously, using MBR and library free search, we increase by 94% the average number of proteins identified per sample compared to a single search run and 60% compared to four sample searches.

Third, we demonstrate that the extracellular vesicles (EVs) plasma fraction can be used as a repertoire of the total content of neat plasma. We purified the EVs fraction by conventional multiple centrifugation, as described previously [20]. We confirmed the enrichment of EVs by the presence of EV markers and organ-specific proteins. Identifying around 4,500 proteins from the EVs fraction is evidence of enrichment in itself, as the abundant proteins must be depleted to achieve this depth. Surprisingly, we found that hundreds of soluble proteins copurify with EVs and that over 90% of the proteins identified in neat plasma were also identified in the EVs fractions. Some of these proteins correlate in intensity, while some are enriched in the EVs fractions, suggesting that they are strongly bound, although possibly non-specifically, to the vesicles.

Analyzing the EVs systematically for each sample would be ideal, as it would allow exceptional depth in plasma analysis. However, this is infeasible with current established purification methods. The preparation requires a large volume of plasma (0.5 mL) and is not scalable or automatable. However, simply by integrating a few EVs files in the analysis of a cohort, the number of proteins identified can be boosted by 15–20%. We showed that using EVs files from 5 healthy donors already improved the analysis of a cohort of neat plasma from unrelated individuals. When possible, we suggest preparing the EVs from a few pools of plasma representative of the cohort analyzed, provided one has enough volume of plasma.

We are aware that MBR works best when samples contain peptides from the same experimental conditions. We verified the identification FDR rate by searching raw files from bacteria alongside neat plasma, and we showed that no additional hits were found above the set 3% FDR, hence confirming the specificity of the additional hits due to EVs files.

In conclusion, we conducted an optimization of neat plasma proteome LC–MS analysis and provide tips that will allow other laboratories to easily improve the depth of the plasma proteome.

All experiments were performed in accordance with the guidelines and regulations described by the Declaration of Helsinki and the Huriet-Serusclat law on human research ethics. The protocol was approved by the international review board of Necker Hospital (NCT01874769, NCT03776474). Informed consent was obtained for all participating subjects.

### Supplementary Information

**Additional file 1: Table S1.** Summary of chromatographic methods.

**Additional file 2: Table S2.** Definition of filters applied in the main DIA-NN report. All filters definition was taken from https://github.com/vdemichev/DiaNN#main-output-reference. Also, it is adviced to use the following q-value filters when using MBR and relying on the main report instead of quantitative matrices: Lib.Q.Value instead of Global.Q.Value. When applying a filter to Q.Value that is more stringent than 1% (e.g. Q.Value < 0.01 filter), always apply the same filter to Lib.Q.Value. Lib.PG.Q.Value instead of Global.PG.Q.Value. These contain normalised quantities for protein groups ('pg_matrix'), gene groups ('gg_matrix'), unique genes ('unique_genes_matrix'; i.e. genes identified and quantified using only proteotypic, that is gene-specific, peptides) and precursors ('pr_matrix'). They are filtered at 1% FDR, using global q-values for protein groups and both global and run-specific q-values for precursors.

**Additional file 3: Fig S1.** Impact of peptide separation: column length and gradient influence. **A** All physico-chemical properties of peptides from the 3 Venn Diagram groups of Fig. 2B (left panel). **B** All physico-chemical properties of peptides from the 3 Venn Diagram groups of Fig. 2B (right panel).

**Additional file 4: Fig S2.** Impact of collection protocol. **A** Number of identified protein groups across 33 neat plasma samples collected using a “single spin” protocol and 5 neat plasma collected using a “double spin” protocol. **B** Contamination ratio across PRP and PPP using coagulation, red blood cell (RBC) and platelets contaminant tracers (from http://plasmaproteomeprofiling.com/)

**Additional file 5: Fig S3. Evaluation of peptides retention on the evotip**. **A** TIC (Total Ion Current) chromatograms of the total plasma sample (input) along with those of the pooled flow-throughs and eluate from the Evotip, onto which the same plasma sample was loaded. **B** Number of identified proteins without (gray) and with (blue) Match Between Runs (MBR) in the inputs, in the combined flow-throughs and the eluates. The experiment was performed in triplicate and the raw files were processed using DIA-NN v1.8.1. **C, D** Overlap of the identified proteins and peptides (70%VV, with MBR) between the inputs, the pooled flow-throughs and the eluates. The Venn diagrams were obtained using Venny 2.1.

## Data Availability

The mass spectrometry proteomics data have been deposited to the ProteomeXchange Consortium via the PRIDE [23] partner repository with the dataset identifier PXD047857.
