# Supplementary material for: Neat plasma proteomics: getting the best out of the worst
Source: Clin Proteomics. 2024 Mar 12;21:22. doi: 10.1186/s12014-024-09477-6 (PMC10935919; doi:10.1186/s12014-024-09477-6)
Supplement: Supplementary file 1 — Additional file 1: Table S1. Summary of chromatographic methods. [file 12014_2024_9477_MOESM1_ESM.pdf]

	Classic 100SPD	Classic 60SPD	Whisper 40SPD	48SPD	24SPD
**LC system**	Evosep	Evosep	Evosep	nElute	nElute
**column**	8 cm performance EV1109 (8cm x 150µm, 1.5µm)	8 cm performance EV1109 (8cm x 150µm, 1.5µm)	IonOpticks Aurora Elite (15cm x 75µm, C18 1.7µm)	IonOpticks Aurora (25cmx75µm, C18 1.6µm)	IonOpticks Aurora (25cmx75µm, C18 1.6µm)
**Flow rate**	1500nL/min	1000nL/min	100nL/min	400nL/min	200nL/min
**Active gradient**	11.5min	21min	31min	12min	30min
**Total gradient**	14.4min	24min	38min	15min	40min
**Column Equilibration time estimated**	2.9min at 2000nL/min	3min at 2000nL/min	8min at 450nL/min	5min (requilibration)+16min (sample loading)	5min (requilibration)+16min (sample loading)
**Sample turnover**	14.4min	24min	38min	36min	61min
