# Supplementary material for: Neat plasma proteomics: getting the best out of the worst
Source: Clin Proteomics. 2024 Mar 12;21:22. doi: 10.1186/s12014-024-09477-6 (PMC10935919; doi:10.1186/s12014-024-09477-6)
Supplement: Supplementary file 2 — Additional file 2: Table S2. Definition of filters applied in the main DIA-NN report. All filters definition was taken from https://github.com/vdemichev/DiaNN#main-output-reference. Also, it is adviced to use the following q-value filters when using MBR and relying on the main report instead of quantitative matrices: Lib.Q.Value instead of Global.Q.Value. When applying a filter to Q.Value that is more stringent than 1% (e.g. Q.Value < 0.01 filter), always apply the same filter to Lib.Q.Value. Lib.PG.Q.Value instead of Global.PG.Q.Value. These contain normalised quantities for protein groups ('pg_matrix'), gene groups ('gg_matrix'), unique genes ('unique_genes_matrix'; i.e. genes identified and quantified using only proteotypic, that is gene-specific, peptides) and precursors ('pr_matrix'). They are filtered at 1% FDR, using global q-values for protein groups and both global and run-specific q-values for precursors. [file 12014_2024_9477_MOESM2_ESM.pdf]

Filters applied	Definition
Q.Value	Run-specific precursor q-value
Lib.Q.Value	q-value for the respective library entry, 'global' if the library was created by DIA-NN. In case of MBR, this applies to the library created after the first MBR pass
Lib.PG.Q.Value	only relevant for MBR: global q-value for the protein group calculated after the first MBR pass
PG.Q.Value	run-specific q-value for the protein group
