# Supplementary figures and images for: Neat plasma proteomics: getting the best out of the worst
Source: Clin Proteomics. 2024 Mar 12;21:22. doi: 10.1186/s12014-024-09477-6 (PMC10935919; doi:10.1186/s12014-024-09477-6)

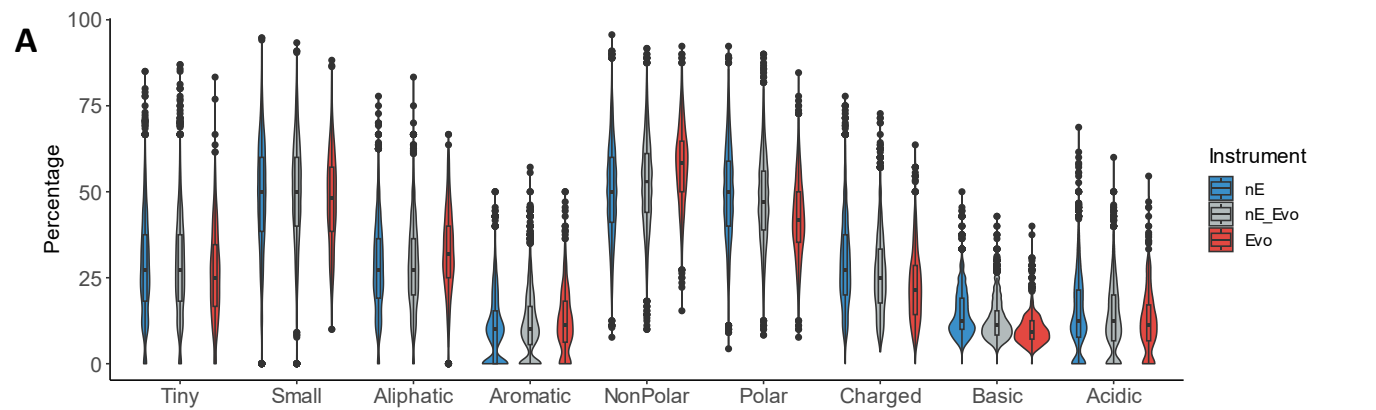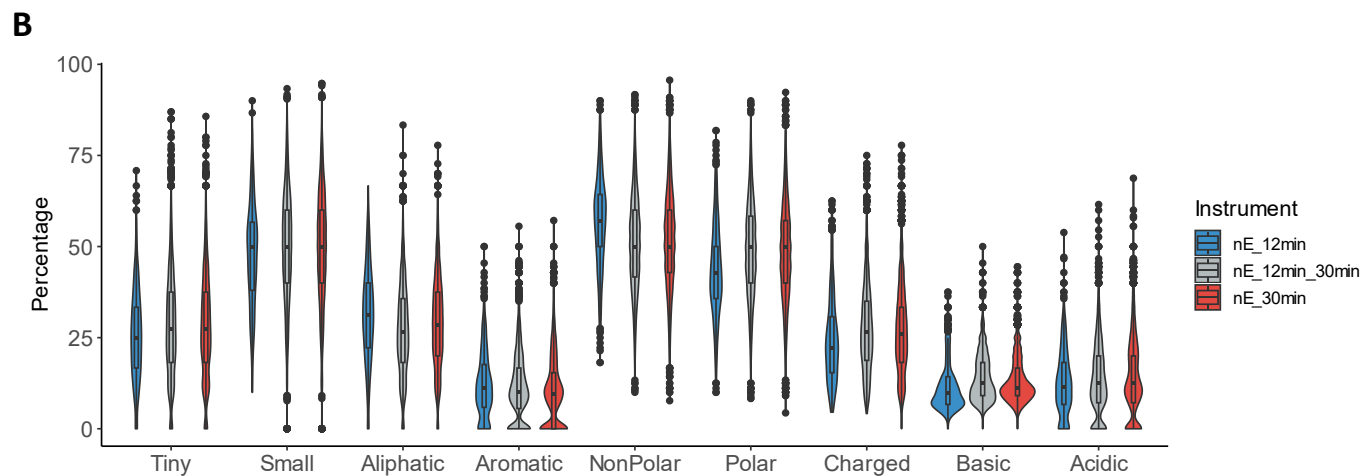

Supplement: Supplementary file 3 — Additional file 3: Fig S1. Impact of peptide separation: column length and gradient influence. A All physico-chemical properties of peptides from the 3 Venn Diagram groups of Fig. 2B (left panel). B All physico-chemical properties of peptides from the 3 Venn Diagram groups of Fig. 2B (right panel). [file 12014_2024_9477_MOESM3_ESM.pdf]

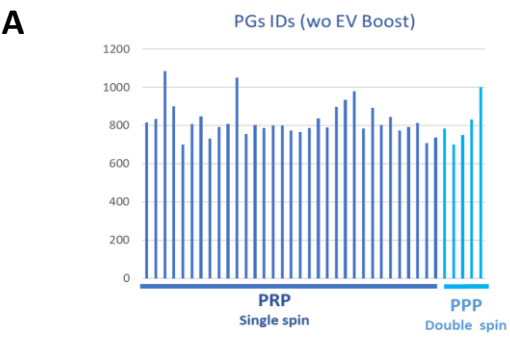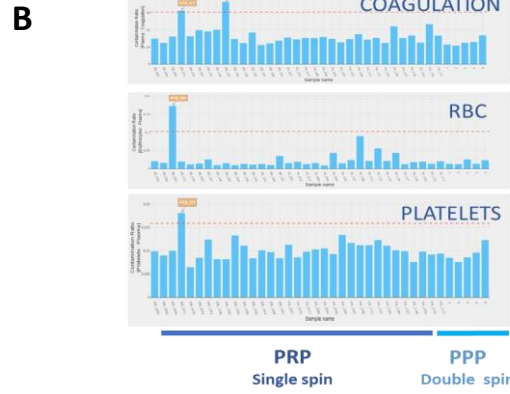

Supplement: Supplementary file 4 — Additional file 4: Fig S2. Impact of collection protocol. A Number of identified protein groups across 33 neat plasma samples collected using a “single spin” protocol and 5 neat plasma collected using a “double spin” protocol. B Contamination ratio across PRP and PPP using coagulation, red blood cell (RBC) and platelets contaminant tracers (from http://plasmaproteomeprofiling.com/) [file 12014_2024_9477_MOESM4_ESM.pdf]

**A**

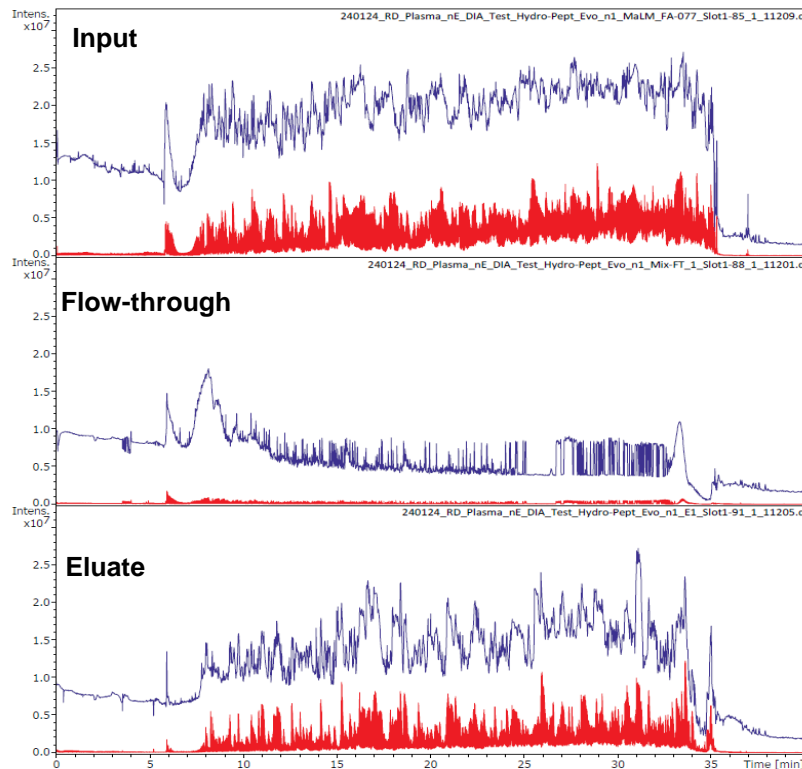

**B**

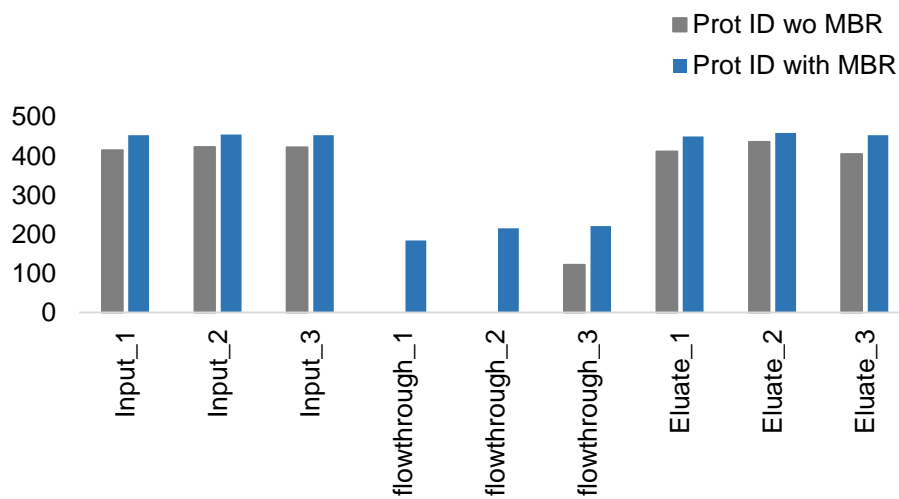

**C**

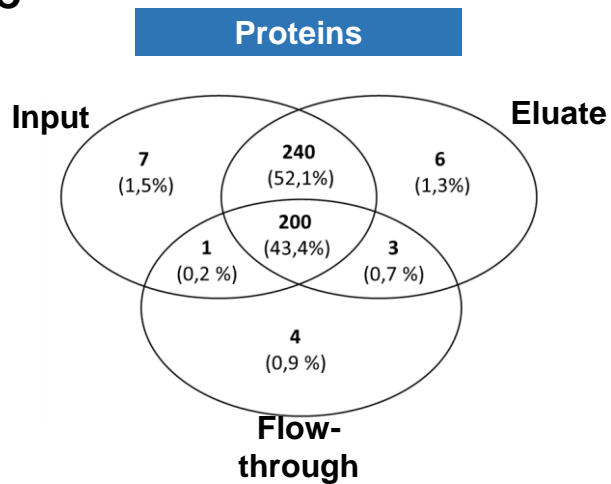

**D**

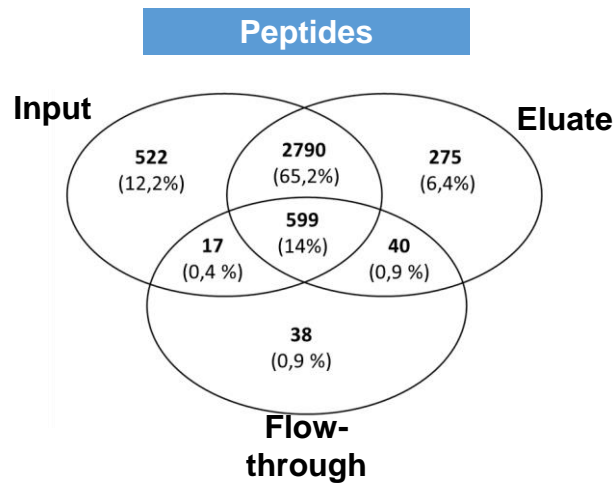

Supplement: Supplementary file 5 — Additional file 5: Fig S3. Evaluation of peptides retention on the evotip. A TIC (Total Ion Current) chromatograms of the total plasma sample (input) along with those of the pooled flow-throughs and eluate from the Evotip, onto which the same plasma sample was loaded. B Number of identified proteins without (gray) and with (blue) Match Between Runs (MBR) in the inputs, in the combined flow-throughs and the eluates. The experiment was performed in triplicate and the raw files were processed using DIA-NN v1.8.1. C, D Overlap of the identified proteins and peptides (70%VV, with MBR) between the inputs, the pooled flow-throughs and the eluates. The Venn diagrams were obtained using Venny 2.1. [file 12014_2024_9477_MOESM5_ESM.pdf]
